# PWR/HDA9/ABI4 Complex Epigenetically Regulates ABA Dependent Drought Stress Tolerance in *Arabidopsis*

**DOI:** 10.3389/fpls.2020.00623

**Published:** 2020-05-26

**Authors:** Irfan Ullah Khan, Akhtar Ali, Haris Ali Khan, Dongwon Baek, Junghoon Park, Chae Jin Lim, Shah Zareen, Masood Jan, Sang Yeol Lee, Jose M. Pardo, Woe Yeon Kim, Dae-Jin Yun

**Affiliations:** ^1^Department of Biomedical Science and Engineering, Konkuk University, Seoul, South Korea; ^2^Division of Applied Life Science, Plant Molecular Biology and Biotechnology Research Center, Gyeongsang National University, Jinju, South Korea; ^3^Instituto de Bioquímica Vegetal y Fotosíntesis, cicCartuja, CSIC-Universidad de Sevilla, Seville, Spain

**Keywords:** *Arabidopsis thaliana*, epigenetic regulation, powerdress (PWR), HDA9, ABI4, chromatin remodeling, deacetylation, drought stress

## Abstract

Drought stress adversely affects plant growth and development and significantly reduces crop productivity and yields. The phytohormone abscisic acid (ABA) rapidly accumulates in response to drought stress and mediates the expression of stress-responsive genes that help the plant to survive dehydration. The protein Powerdress (PWR), which interacts with Histone Deacetylase 9 (HDA9), has been identified as a critical component regulating plant growth and development, flowering time, floral determinacy, and leaf senescence. However, the role and function of PWR and HDA9 in abiotic stress response had remained elusive. Here we report that a complex of PWR and HDA9 interacts with ABI4 and epigenetically regulates drought signaling in plants. T-DNA insertion mutants of *PWR* and *HDA9* are insensitive to ABA and hypersensitive to dehydration. Furthermore, the expression of ABA-responsive genes (*RD29A*, *RD29B*, and *COR15A*) is also downregulated in *pwr* and *hda9* mutants. Yeast two-hybrid assays showed that PWR and HDA9 interact with ABI4. Transcript levels of genes that are normally repressed by ABI4, such as *CYP707A1*, *AOX1a* and *ACS4*, are increased in *pwr*. More importantly, during dehydration stress, PWR and HDA9 regulate the acetylation status of the *CYP707A1*, which encodes a major enzyme of ABA catabolism. Taken together, our results indicate that PWR, in association with HDA9 and ABI4, regulates the chromatin modification of genes responsible for regulation of both the ABA-signaling and ABA-catabolism pathways in response to ABA and drought stress.

## Introduction

During their life cycles, plants are continuously exposed to environmental challenges including light, heat, cold, flooding, high salinity, and drought stress. Among them, drought stress results in considerable damage to plant growth, and more than 40% of crop production is lost to drought (Guy et al., 1985; May et al., 1998; Hasegawa et al., 2000). Upon exposure to drought stress, plants initiate the expression of resistance genes and subsequent activation of signaling pathways. Plants have developed complex molecular and signaling mechanisms to adapt to water deficit condition. They respond to drought stress either through osmotic adjustment and regulation of ion homeostasis or by controlling the damage repair system and the detoxification and removal of reactive oxygen species (ROS) (Shinozaki and Yamaguchi-Shinozaki, 2000; Zhu, 2002). The phytohormone abscisic acid (ABA) plays a crucial role in plant physiological processes, regulating many aspects of plant growth and development including seed dormancy, seed maturation, and seedling growth. ABA is also required for drought stress tolerance, which regulates stomatal movement during drought stress and helps plants tolerate extreme water-deficient conditions (Finkelstein and Lynch, 2000; Lopez-Molina et al., 2001). However, the molecular and biochemical mechanism of these signaling pathways are not yet fully understood.

The mechanisms of chromatin modification and the constitution of chromatin complexes regulating gene expression are highly conserved in plants, mammals and yeast (Henderson and Jacobsen, 2007). Chromatin structure and its modification form the basic mechanism of genetic and epigenetic regulation of stress-related gene expression (Horn and Peterson, 2002). Histone acetylation is one of the most important features of chromatin remodeling, which removes positive charges by adding an acetyl group to the lysine residues of histone proteins, thereby reducing the histone–DNA affinity and resulting in chromatin decondensation and active transcription (Bannister and Kouzarides, 2011). Histone acetylation levels are dynamically regulated through the combined actions of histone acetyltransferases (HATs) and histone deacetylases (HDACs). HDACs are enzymes conserved in the eukaryotes that function in biological processes including transcription, genome stability, development and in biotic and abiotic stress responses (Haberland et al., 2009; Seto and Yoshida, 2014; Bosch-Presegué and Vaquero, 2015).

Human NCOR1 is a homolog of the *Arabidopsis* protein powerdress (PWR), which is involved in the floral determinacy network (Yumul et al., 2013). The gene encoding PWR was named *Powerdress* because of the appearance of the single mutant, which has bulged carpel tips reminiscent of excessively padded suit or dress shoulders. PWR has two conserved SWI3/DAD2/N-CoR/TFIII-B (SANT) domains that together function as a histone-interaction module that couples histone tail binding to enzyme catalysis for the remodeling of nucleosomes. PWR interacts with REDUCED Potassium Dependency Protein 3 (RPD3), a class-1-type Histone Deacetylase 9 (HDA9) and mediates Histone 3 (H3) deacetylation. The complex of PWR*–*HDA9 also regulates flowering time in *Arabidopsis* by repressing *Agamous-Like 19* (*AGL19*) transcription (Kim et al., 2016).

HDA9 requires PWR for its nuclear transport and binding to the promoter region of key negative regulator genes involved in leaf senescence (Yumul et al., 2013; Chen et al., 2016). HDA9 interacts with PWR and WRKY53 to regulate leaf aging, and *hda9* and *pwr* loss-of-function mutants exhibit late senescence phenotype (Chen et al., 2016). Furthermore, ABA promotes leaf senescence and loss of function of its receptors PYL8 and PYL9, resulting in delayed leaf senescence (Zhao et al., 2015). To date, approximately 18 *Arabidopsis* histone deacetylases (HDACs) have been identified. These are divided into three main types. Twelve belong to the reduced Potassium Dependency Protein 3/HDA1 Histone Deacetylase 1 (RPD3) superfamily and are named as HDAs; two are in the histone deacetylase 2 (HD2) family and are named HDTs; and two belong to the silent information regulator protein 2 (SIR2) family and are named SRTs (Pandey et al., 2002; Hollender and Liu, 2008). RPD3-type class 1 HDA6 and HDA19 are involved in the regulation of seed germination, ABA response, salt stress and other abiotic stresses. Unlike *hda6* and *hda19* mutants, mutants of *HDA9* (*hda9-1* and *hda9-2*) are insensitive to ABA and to salt stress during seed germination and root growth (van Zanten et al., 2014; Kang et al., 2015).

Plant endogenous ABA concentration is determined by the rate of ABA metabolism (i.e., biosynthesis, and catabolism). The molecular mechanisms of ABA signaling, biosynthesis, and catabolism have been characterized using genetic and biochemical approaches (Nambara, 2005). *Arabidopsis* contains several *NCED* family genes, including *AtNCED3*, which plays the central role in ABA biosynthesis in response to drought stress (Iuchi et al., 2001). The transcript level of *AtNCED3* rapidly increases in response to drought stress, while a *nced3* mutant carrying a T-DNA insertion is defective in accumulation of endogenous ABA under drought stress and impaired in drought stress tolerance. For ABA catabolism, at least two crucial pathways have been characterized: the oxidative pathway and the sugar-conjugation pathway (Nambara, 2005). The oxidative pathway is stimulated by *CYP707A*-induced hydroxylation of ABA C-80 to phaseic acid (Kushiro et al., 2004; Saito et al., 2004). The four *Arabidopsis CYP707A*-family genes encoding ABA 8′-hydroxylases are induced by exogenous ABA, as well as by dehydration and other abiotic stresses (Kushiro et al., 2004; Saito et al., 2004). By contrast, *CYP707A2* transcripts predominantly accumulate in dry seeds, and the gene is immediately upregulated after seed imbibition. The *cyp707a2* mutant maintains a high level of ABA and exhibits enhanced seed dormancy as compared to the wild type (WT) (Kushiro et al., 2004). These reports indicate that *CYP707A2* is a component in ABA catabolism during seed germination and regulation of seed dormancy. However, the physiological role of other *CYP707A* genes remained unclear.

The interaction and binding of ABA with PYL/PYR1/RCAR receptors results in the deactivation of protein phosphatase type-2C (PP2C) proteins (*ABI1*, *ABI2*, *HAB1* and *HAB2*), thereby releasing SNF1-related protein kinase (SnRK2) kinases. The ABA-mediated disassociation of PP2C from SnRK2s leads to autophosphorylation and subsequently to transphosphorylation, activation of the downstream targets (such as ABI3, ABI4, and ABI5) and ultimately regulation of downstream signaling pathways (Fujii et al., 2009; Umezawa et al., 2009; Antoni et al., 2012). ABI4 is an important transcription factor that was initially identified as a member of the AP2/ERF family and that binds to ABA-responsive *cis*-regulatory elements (CREs), ABRE and regulates the expression of genes in response to abiotic stresses (Mizoi et al., 2012). ABI4 is also a versatile activator and a repressor of several genes. Beside inducing the expression of genes involved in seed dormancy, ABA signaling, salt stress and floral transition (Söderman et al., 2000; Nakabayashi et al., 2005; Koussevitzky et al., 2007; Bossi et al., 2009; Giraud et al., 2009; Reeves et al., 2011; Shu et al., 2018), ABI4 also represses the expression of genes involved in ABA catabolism (*CYP707A* genes), ethylene biosynthesis (*ACS* genes) and retrograde signaling (*AOX1a*), genes encoding *Arabidopsis* response regulators (*ARRs*) (Huang et al., 2017), as well as genes involved in fatty acid biosynthesis, photosynthesis, pigment and wax metabolic processes (Shu et al., 2013; Dong et al., 2016), by directly binding to their promoters.

PWR also interacts with HOS15 (HIGH EXPRESSION OF OSMOTICALLY RESPONSIVE GENES 15), a homolog of human transducin-β-like protein 1 (TBL1). HOS15 contains a LisH and a WD40-repeat domain and is involved in histone modification and deacetylation during abiotic stresses. Furthermore, HOS15 also interacts with HISTONE DEACETYLASE 9 (HDA9), as determined by affinity purification of HOS15-interacting proteins (Park et al., 2018). Loss-of-function *hos15-2* mutant plants are hypersensitive to ABA during germination and extremely tolerant to drought stress, indicating the importance of HOS15 as a negative regulator (Ali et al., 2019). On the other hand, the function of PWR in abiotic stresses is largely unknown.

Here we report that T-DNA insertion mutants of PWR (*pwr-2* and *pwr-3*) are ABA insensitive and display drought-sensitive phenotypes. Using yeast two-hybrid screening, we observed that both PWR and HDA9 interact with ABI4 along with ABI3. The expression of ABA-responsive genes is downregulated in *pwr* and *hda9* mutants. Transcript levels of genes that are normally repressed by ABI4, such as *CYP707A* genes, *AOX1a* and *ACS4*, are upregulated in the *pwr* and *hda9* mutants. Moreover, in response to drought stress, PWR and HDA9 regulate acetylation at the promoter of *CYP707A1*, which encodes the major enzyme of ABA catabolism. Taking these results together, we conclude that PWR in association with HDA9 and ABI4 regulates the chromatin modification of genes responsible for ABA catabolism in response to drought stress.

## Materials and Methods

### Plant Materials and Growth Conditions

Plants from the *Arabidopsis thaliana* ecotype Columbia-0 (Col-0) background were used in this study. Seeds of the WT and mutants were surface sterilized in a solution containing 3% sodium hypochlorite solution (Yakuri Pure Chemicals, Kyoto, Japan) for 5 min and then rinsed five times with sterilized water. After stratification for 3 day at 4°C in the dark, the plants were grown on half-strength Murashige and Skoog (1/2 MS) medium or soil at 23°C under a 16-h light/8-h dark condition. The T-DNA insertion mutant *pwr-2* (SALK_0718811C) seeds were obtained from ABRC stock center and previously described by Yumul et al. (2013) and the mutant *pwr-3* (SALK_006823), was also obtained from ABRC stock center. The T-DNA insertions in these plants were confirmed by genotyping PCR. The *hda9-1* (Gk_305G03) and *hda9-2* (SALK_007123) mutants were obtained from NASC^1^ and ABRC^2^, respectively (Alonso et al., 2003; Rosso et al., 2003; Kang et al., 2015). The *pwr-2/hda9-1* double-mutant plants were created by crossing.

### RNA Extraction and Quantitative PCR Analysis

Total RNAs (2 μg) from plants (harvested at the time points described in the text for each experiment) were extracted with the RNeasy Plant Mini Kit (Qiagen, Hilden, Germany), treated with DNase I (Sigma, St. Louis, MO, United States) and used to synthesize first-strand cDNA using the Thermoscript^TM^ RT-PCR System (Invitrogen, Carlsbad, CA, United States). Quantitative RT-PCR (qRT-PCR) was performed using SYBR Green PCR Master Mix kit (Bio-Rad SYBR Green Supermix, Hercules, CA, United States) according to the manufacturer’s instructions with the CFX96 or CFX384 Realtime PCR detection system (Bio-Rad, CA, United States). The relative expression levels were calculated using the comparative cycle threshold (2^–ΔΔ*CT*^) method. The sequences of the primers used in qRT-PCR are listed in Supplementary Table S1.

### Physiological and Phenotype Assay

For ABA germination assays, seeds were grown on 1/2 MS medium containing 1.5% sucrose and different concentrations of ABA (Sigma, St. Louis, MO, United States). Successful germination in the presence of ABA was determined by germination rate and the presence of green cotyledons at the indicated concentrations. For drought test, 3-week-old plants were subjected to drought stress by withholding water for 14 day while control plants were watered as before. The drought-stressed plants were then re-watered, and their recovery was monitored. Three experimental repeats were carried out, each involving at least 36 plants from each category.

### Plasmid Constructions

The full-length *PWR*, *HDA9* and *ABI4* coding sequences were amplified with the primers listed in Supplementary Table S1 to generate the entry vector [*PWR*, *HDA9*, *ABI4* with or without stop codons in the *pDONRTM/Zeo* vector (Invitrogen, Carlsbad, CA, United States)]. *pDONRTM/Zeo-HDA9* and *pDONRTM/Zeo-ABI4* were fused into the *pGWB14* destination vectors by *in vitro* recombination using Gateway BP and LR reaction kits (Invitrogen, Carlsbad, CA, United States) to generate *HDA9-3xHA*. *ABI4* was cloned into the *pK7WGF* destination vector to construct *GFP-ABI4*. The specific primer sequences are provided in Supplementary Table S1.

### Yeast Two-Hybrid Assay

For yeast two-hybrid experiments, *pDONRTM/Zeo-PWR* and *pDONRTM/Zeo*-HDA9 were fused into the yeast two-hybrid destination vector *pDEST22* (harboring the activation domain) and *pDONRTM/Zeo*-ABI4 was fused into the destination vector *pDEST32* (harboring the DNA binding domain) to generate the construct vectors *pDEST22-PWR*, *pDEST22-HDA9, and pDEST32-*ABI4, respectively. These plasmids were transformed into the *Saccharomyces cerevisiae* strain PJ-694-A. Individual colonies of transformants were streaked on agar plates containing synthetic complete (SC) medium lacking tryptophan and leucine, and then grown for 48 h. The interaction of PWR, HDA9, and ABI4 was tested on plates containing medium without histidine and further tested in growth medium containing 3-amino-1,2,4-triazole (3-AT). Empty vector was used as a negative control, while the combination of *pDEST22-SOS2* and *pDEST32-SOS3* was used as positive control.

### Nuclear-Cytoplasmic Fractionation Assay

Nuclear proteins were extracted from 2-week-old seedlings treated with dehydration stress for indicated time point by CELLYTPN1 CelLytic PN Isolation/Extraction Kit (Sigma-Aldrich), crude preparation. Anti-H3 (Abcam) and anti-AcH3 (Millipore) antibodies and antigen proteins were visualized by chemiluminescence using ECL detecting reagent (Bio-Rad).

### Co-Immunoprecipitation Assay

For co-immunoprecipitation assays, *35S:ABI4-GFP* and *35S:HDA9-HA* expression cassettes were co-infiltrated into leaves of *N. benthamiana*, and after 3 day of incubation, total protein was extracted from the leaves, pulled down with α-GFP, and immunoblotted with α-HA. Each immunoblot was incubated with the appropriate primary antibody (α-HA antibody, 1:2000; α-GFP antibody) for 2 h at room temperature or overnight at 4°C. The membranes were developed using peroxidase-conjugated secondary antibody: 1:2000 for α-rabbit antibody (GE, Little Chalfont, Buckinghamshire, United Kingdom) and 1:1000 for α-rat IgG (Sigma, St. Louis, MO, United States).

### Chromatin Immunoprecipitation (ChIP) Assay

ChIP assays were carried out following an established method as previously described (Saleh et al., 2008). Two-week-old control and dehydration-treated *Arabidopsis* plants were treated with 1% formaldehyde for 15 min to fix the chromatin structure and this cross-linking reaction was subsequently stopped by treatment with 0.1 M glycine for 5 min. The DNA-fixed plant tissues were ground with liquid nitrogen and washed with water and then the nuclei were isolated. Nuclear proteins were extracted and sonicated with a Bioruptor (BMS) to fragment the chromosomal DNA. Immunoprecipitation was performed using an antibody to total anti-acetylated H3 (Millipore), with salmon sperm DNA and protein A agarose (upstate Biotechnology).

### Measurement of Stomatal Aperture

Leaves of 12-day-old seedlings were floated on stomatal opening buffer (5 mM 2-(N-morpholino) ethanesulfonic acid [MES], 5 mM KCl, 50 mM CaCl^2^ [pH 5.6]) under light for 3 h. After 5 μM ABA treatment for 2 h, leaves were fragmented in a warning blender. Samples were rinsed with pure water three times for 10 min each. Washed samples were incubated over–night in the secondary fixative solution, 2% OsO^4^, in the dark at 4C. After fixation, OsO^4^ was removed by washing the samples three times for 10 min each. The samples were then dehydrated chemically for embedding in a series of EtOH solutions: 20, 50, 70, 90%, and finally 100% EtOH sequentially for 40 min each. Epidermal fragments were quickly mounted for scanning electron microscopy (SEM) (JSM-6380LV; JEOL, Eching, Munchen, Germany) assay. At least 10 stomata from three different plants of each genotype were used to measure the stomatal aperture with three biological repeats. Each experiment was replicated three times.

## Results

### Mutations in PWR Reduces ABA Responsiveness in *Arabidopsis*

**FIGURE 1:**
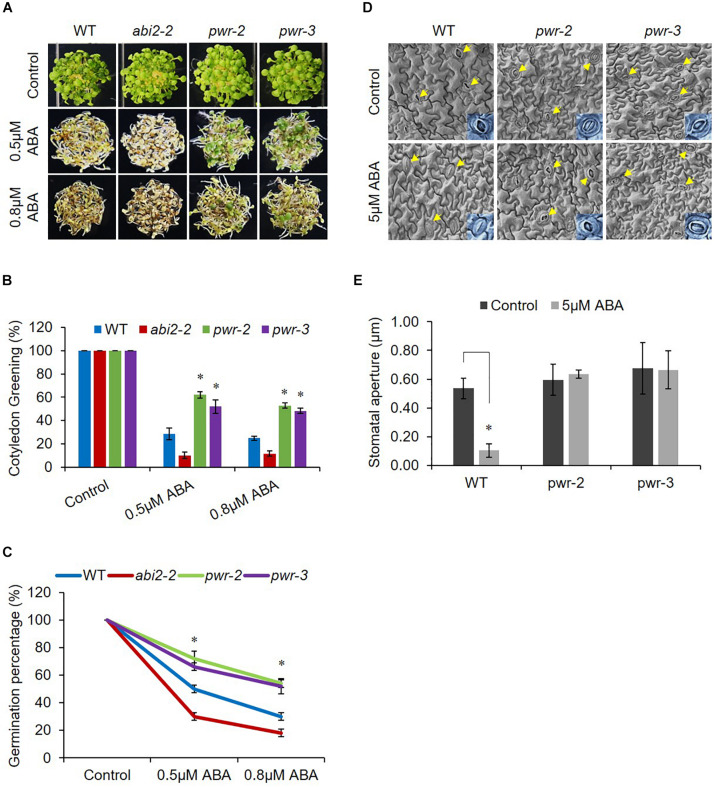
*pwr* mutants are insensitive to ABA. **(A)** WT, *abi2-2*, *pwr-2*, and *pwr-3 Arabidopsis* seeds were germinated on 1/2 MS medium supplemented with 0.5 μM and 0.8 μM ABA (or without ABA for controls). Photographs were taken 7 day after germination. **(B)** The numbers of green cotyledons from each line were counted after 7 day. Mean ±SD values were determined from three replicates (*n* = 144). Significant difference was determined by Student *t*-test (^∗^*p* < 0.01). **(C)** Germination rates of indicated genotypes from **(A)**. Mean ±SD values were determined from three replicates (*n* = 144). Significant difference was determined by Student *t*-test (^∗^*p* < 0.01). **(D)** *pwr* mutation impairs ABA-induced stomatal closure. Seedlings of WT (Col-0) and *pwr* mutants were exposed to 2-h ABA (5 μM) treatment. Epidermal peels from WT, *pwr-2*, and *pwr-3* were measured for stomatal aperture in control condition and in response to ABA (arrows indicate stomata). **(E)** Quantitative analysis of **(D)** using Image J 1.47V software. At least 10 stomata from three different plants of each genotype were used to measure stomatal aperture. Error bars represent SE. Significant difference was determined by a Student’s *t*-test (^∗^*p* < 0.01).

Powerdress regulates plant growth and developmental processes (Yumul et al., 2013; Chen et al., 2016; Kim et al., 2016); however, we were interested in assessing its involvement in abiotic stresses. Therefore, to investigate PWR possible involvement in ABA signaling, we tested the physiological response of *pwr-2* and *pwr-3* to exogenously applied ABA. We germinated seeds of WT (Col-0), *pwr-2* and *pwr-3* lines, as well as the loss-of-function *abi2-2* mutant (which is hypersensitive to ABA) as an experimental control, on Murashige and Skoog (MS) medium containing ABA. In the presence of ABA, *pwr-2* and *pwr-3* seedlings exhibited greater germination than WT seedlings, and *abi2-2* seedlings showed even poorer germination and cotyledon greening (Figures 1A–C). After exposure to 0.5 μM ABA for 7 days, the percentages of green cotyledons for *pwr-2* and *pwr-3* were 62 and 52–55% respectively, compared with 27–30% for WT and 10–15% for *abi2-2* while, percentages of green cotyledons for *pwr-2* and *pwr-3* were 53 and 49% respectively as compared to 25% of WT on 0.8 μM ABA (Figure 1B). Since PWR loss-of-function mutants displayed ABA-insensitive phenotypes, it seemed likely that PWR might play a role in regulating plant response to drought. To test this hypothesis, we exposed 3-week-old WT, *abi2-2*, *pwr-2*, and *pwr-3* plants to 14 days of drought stress. Plants were re-watered after the drought period and their survival rates recorded 2 days after re-watering. The WT and *abi2-2* plants survived the dehydration stress at rates of 80% and 100%, respectively (Supplementary Figure S1A). By contrast, *pwr-2* and *pwr-3* mutants were unable to tolerate water-deficient condition and survived at rates of only 10 and 15%, respectively (Supplementary Figure S1B). Furthermore, *pwr* mutants showed impaired stomatal closure in leaf epidermal fragments after treatment with exogenous ABA (Figures 1D,E), indicating that the drought sensitivity of *pwr* mutants is correlated with reduced stomatal closure. Taken together these results indicating that PWR plays a central role in plant sensitivity to ABA in seed germination and confers tolerance of drought.

### PWR and HDA9 Work Together in the Same Pathway

**FIGURE 2:**
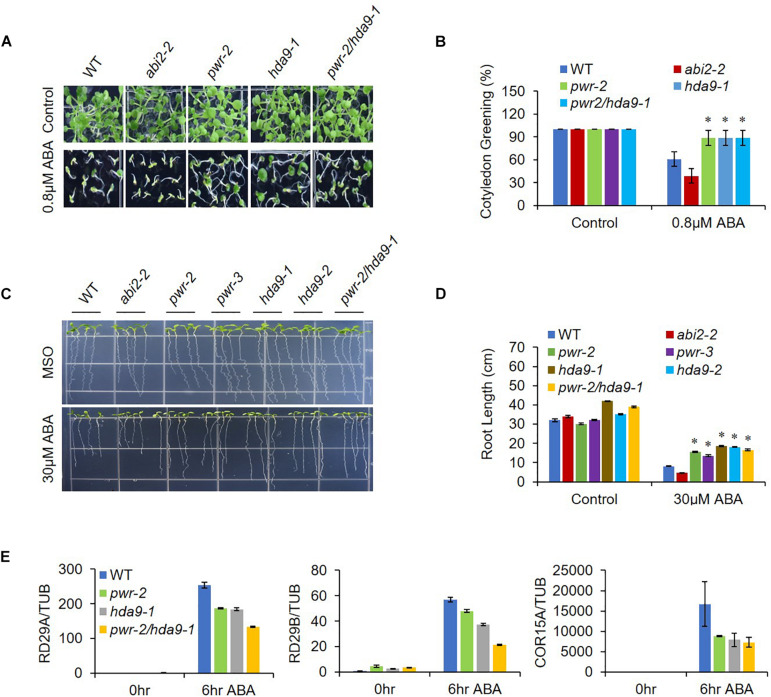
*pwr* and *hda9* mutants are insensitive to ABA. **(A)** Seeds WT, *abi2-2*, *pwr, hda9*, and *pwr/hda9* double mutant were germinated on 1/2 MS medium with 0.8 μM ABA. Photographs were taken 7 day after germination. **(B)** Numbers of green cotyledons were counted after 7 day. Mean ±SD values were determined from three replicates (*n* = 45). Significant difference was determined by Student *t*-test (^∗^*p* < 0.01). **(C)** Root growth phenotypes of WT, *abi2-2*, *pwr-2*, *pwr-3*, *had9-1*, *hda9-2*, and *pwr-2/hda9-1*. Photographs were taken 10 day after transfer of 4-day-old seedlings to MS medium with or without 30 μM ABA. **(D)** Quantification of primary root lengths of the seedling depicted in C. Error bars represent means ± SE of three replicates (*n* = 20 seedlings per replicate). Significant difference was determined by Student *t*-test (^∗^*p* < 0.01). **(E)** Expression of three ABA-responsive stress-related genes (*RD29A*, *RD29B*, and *COR15A*) in WT, *pwr-2*, *hda9-1*, and *pwr-2/hda9-1 Arabidopsis* during ABA stress was analyzed by qRT-PCR. The imbibed seeds were germinated and grown on MS medium for 10 days, and then the seedlings were harvested and treated with 50 μM ABA to obtain cDNA for qRT-PCR. Samples were collected at the indicated time points: 0 h (no treatment) and 6 h. Total RNAs were extracted from those seedlings, and RT-qPCR analysis was performed. *TUB4* was used as internal control. Error bars show SD.

**FIGURE 3:**
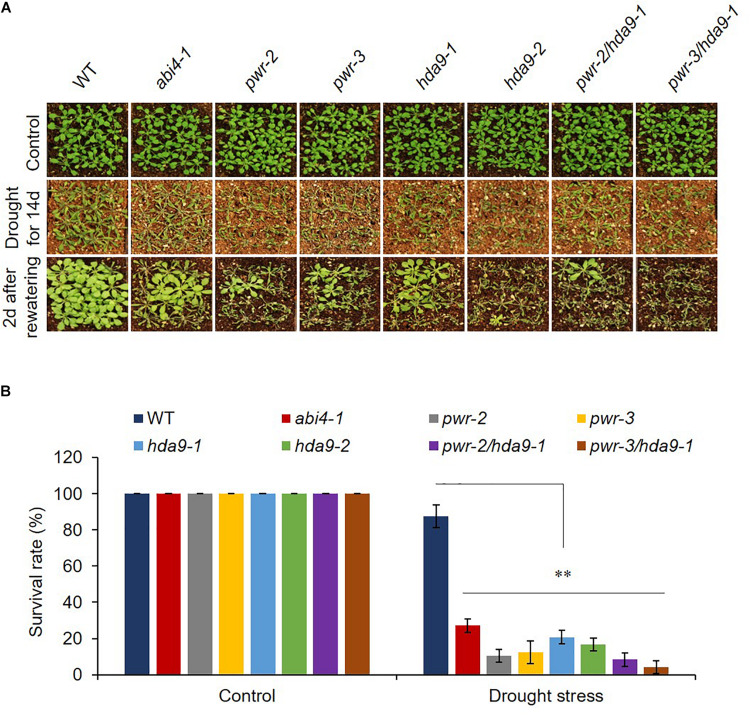
*pwr* and *hda9* mutant plants are drought sensitive. **(A)** 3-week-old plants of genotypes WT, *abi4-1*, *pwr, hda9* single, and *pwr/hda9* double mutants exposed to drought assay by withholding water for 14 days and subsequently rewatered the plants after drought period. Photographs were taken 2 days after rewatering. **(B)** Survival rate (percentage) of genotypes shown in **(A)**. Results are means of three independent biological repeats. Significant difference was determined by Student *t*-test (^∗∗^*p* < 0.001).

As previously described, HDA9 represses the seedling trait and negatively regulates salt and drought stress tolerance; in addition, HDA9 requires PWR for its nuclear transport and promoter association. Furthermore, PWR and HDA9 also regulate flowering through repression of *AGL19* (van Zanten et al., 2014; Kang et al., 2015; Kim et al., 2016; Zheng et al., 2016). In this context, we were interested in investigating whether PWR and HDA9 also work together in response to the phytohormone ABA. We therefore tested the physiological response of PWR and HDA9 mutants to exogenous ABA. Compared to WT, *pwr*, *hda9*, and *pwr/hda9* double-mutant seedlings were relatively insensitive to exogenous ABA (Figure 2A). In the presence of 0.8 and 0.5 μM ABA (in Supplementary Figure S2), the percentages of green cotyledons for *pwr-2*, *pwr-3*, *hda9-1*, *hda9-2*, and *pwr-2/hda9-1* were approximately 83, 85, 82, 84, and 85%, respectively, compared with 55% for WT, 35% for *abi2-2* and 98% for *abi4-1* (Figure 2B and Supplementary Figure S2). To test root growth phenotypes in the presence of ABA, we transferred 4-days-old seedlings of WT (Col-0), *abi2-2*, *pwr2*, *pwr-3*, *hda9-1*, *hda9-2*, and *pwr-2/hda9-1 Arabidopsis* to MS medium containing 30 μM ABA and allowed them to grow for a further 10 days. Compared to that of the WT, the root growth of single and double mutants of PWR and HDA9 was insensitive to ABA (Figure 2C). The relative reductions in root length were 37.5–43.3% for *pwr*, 42.8–45.7% for *hda9* and 39.4% for the *pwr-2/hda9-1* double mutant, as compared to 70 and 85%, respectively, for WT and *abi2-2* (Figure 2D). Consistent with phenotypes, the induction of ABA-responsive genes, including *RD29A*, *RD29B*, and *COR15A*, upon ABA was lower in *pwr-2*, *hda9-1* and *pwr-2/hda9-1* than in WT plants (Figure 2E). Moreover, we also tested the dehydration responses of PWR and HDA9 mutants by exposing 3-week-old plants to 14-days drought stress, re-watering them and then recording the survival rate 2 days later. As expected, like the *pwr-2* and *pwr-3* mutants, *hda9* mutants were extremely sensitive to drought stress (Figure 3). Taken together, these results suggested that PWR and HDA9 work together and play a critical role in plant ABA response and drought tolerance.

### PWR and HDA9 Physically Interact With ABI4

**FIGURE 4:**
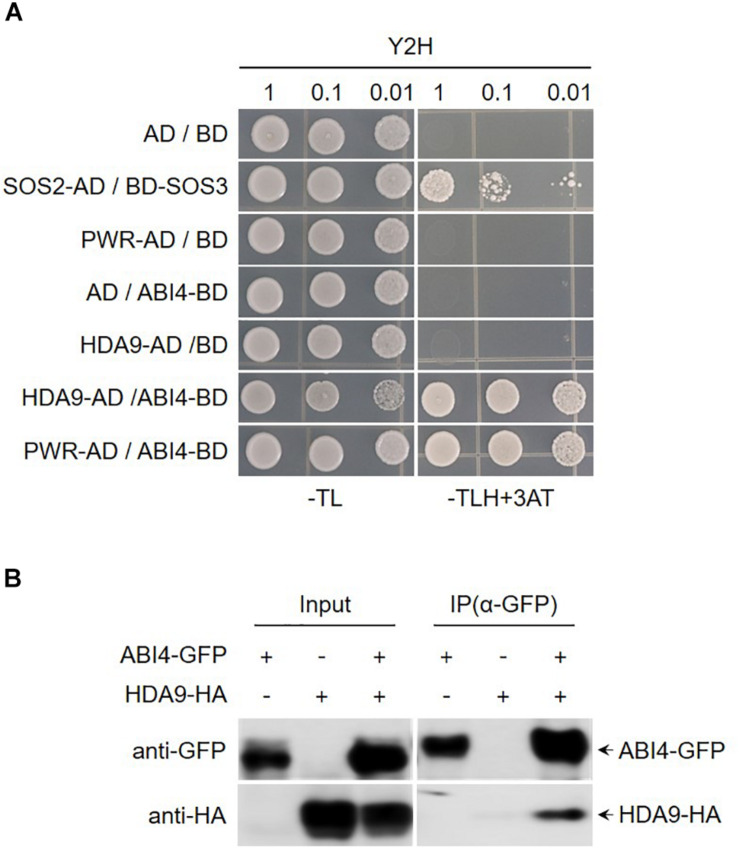
PWR and HDA9 physically interact with ABI4. **(A)** Screening of PWR and HDA9 with ABA transcription factors using yeast two-hybrid assay. BD, *pDEST32* (bait plasmid); AD, *pDEST22* (prey plasmid). The co-transformed yeast strains were plated on the control −TL medium and selective −TLH + 3-AT medium. The combinations with empty plasmid were used as negative controls and SOS2-AD/SOS3-BD was used as positive control. **(B)** Co-immunoprecipitation assay between HDA9 and ABI4. Protein extracts obtained from *N. benthamiana* leaves infiltrated with *Agrobacterium tumefaciens* harboring *35S:HDA9-HA* and *35S:ABI4-GFP* were analyzed using anti-GFP and anti-HA antibodies. Protein extracts (input) were immunoprecipitated (IP) with anti-GFP. Immunoblots were analyzed with anti-GFP and anti-HA to detect interaction between HDA9 and ABI4.

*Arabidopsis* HDA9 regulates several aspects of biological processes such as seed dormancy and maturation, flowering time, and stress responses. Moreover, previous reports have shown that developmental and stress-related genes are hyperacetylated and upregulated in *hda9* mutant. The direct interaction between PWR and HDA9 and the similar type of molecular and morphological defects in *pwr* and *hda9* mutants strongly suggest that PWR and HDA9 are working together in same complex (Kim et al., 2013; van Zanten et al., 2014; Kang et al., 2015; Lee et al., 2016; Zheng et al., 2016). To investigate the interaction of proteins with PWR and HDA9 that are involve in seed germination and response to drought stress, we carried out yeast two-hybrid screening of ABA- and drought-stress-responsive transcription factors, and we observed that PWR and HDA9 specifically interact with ABI3 and ABI4 (Figure 4A and Supplementary Figure S3). To validate the interaction between ABI4 and the HDA9-PWR complex, we performed a co-immunoprecipitation assay using *Nicotiana benthamiana* leaves transiently expressing 35S:HDA9-HA and 35S:ABI4-GFP. ABI4-GFP successfully pulled down HDA9-HA, indicating that ABI4 forms a complex with HDA9 and PWR (Figure 4B).

### Genes Repressed by ABI4 Are Upregulated in *pwr* Mutants During Dehydration

**FIGURE 5:**
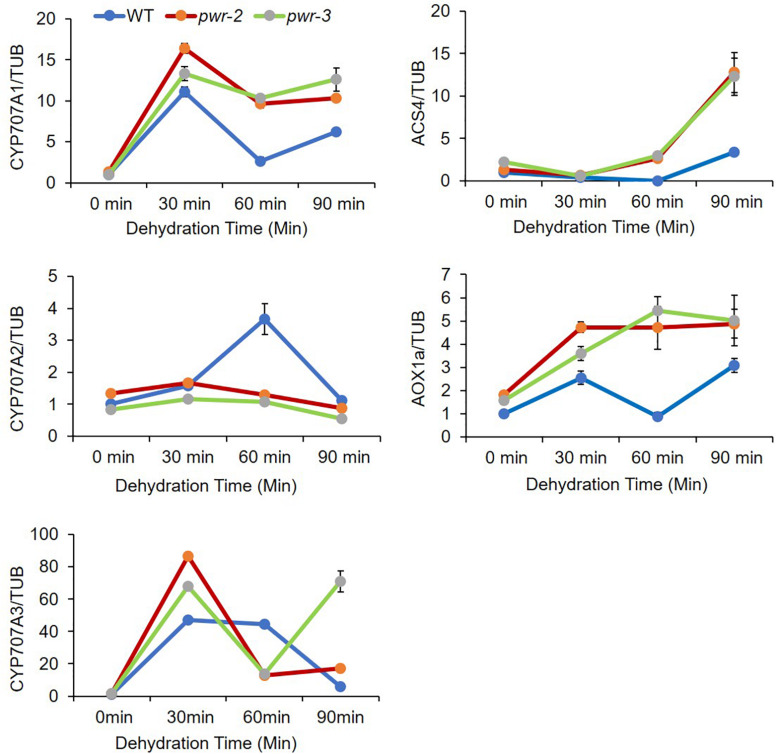
PWR negatively regulates ABI4-responsible genes. The relative expression of ABA hydroxylase genes analyzed by qRT-PCR. 10-day-old seedlings of WT, *pwr-2*, and *pwr-3* were dehydrated at room temperature for indicated time point. Total RNAs were extracted from those seedlings, and RT-qPCR was performed to analyze the abundance of *CYP707A1*, *CYP707A2*, *CYP707A3, ACS4*, and *AOX1a* expression. Expression levels were normalized to that of *TUB4*. Normalized expression is shown as mean ± standard deviation (SD). The experiment was repeated three times.

ABI4 is identified as a member of the AP2/ERF superfamily that binds specifically to ABRE elements and regulates abiotic-stress-related gene expression (Mizoi et al., 2012). ABI4 plays dual function in regulating gene expression, serving both as an activator and as a repressor (Söderman et al., 2000; Nakabayashi et al., 2005; Koussevitzky et al., 2007; Bossi et al., 2009; Giraud et al., 2009; Reeves et al., 2011; Shu et al., 2018). To identify the transcriptional regulatory function of PWR in drought stress response, we monitored the expression of genes repressed by ABI4 in *pwr* mutants treated with different time point of dehydration stress. Surprisingly, the expression of ABA hydroxylase gene *CYCP707A1* was upregulated in *pwr-2* and *pwr-3* mutants as compared with WT (Figure 5), although the expression of other *CYCP707A* genes were unchanged. Similarly, the expression of other ABI4 target genes such *AOX1a* (a retrograde signaling gene) and *ACS4* (an ethylene biosynthesis genes) in *pwr-2* and *pwr-3* in response to dehydration stress was also high (Figure 5). These results indicated that ABI4 alone is not enough to suppress these genes and that ABI4 requires the PWR and HDA9 repressor complex to target different genes in regulating plant stress tolerance.

### PWR and HDA9 Regulate Acetylation Level at *CYP707A1* Promoter Under Drought Stress

**FIGURE 6:**
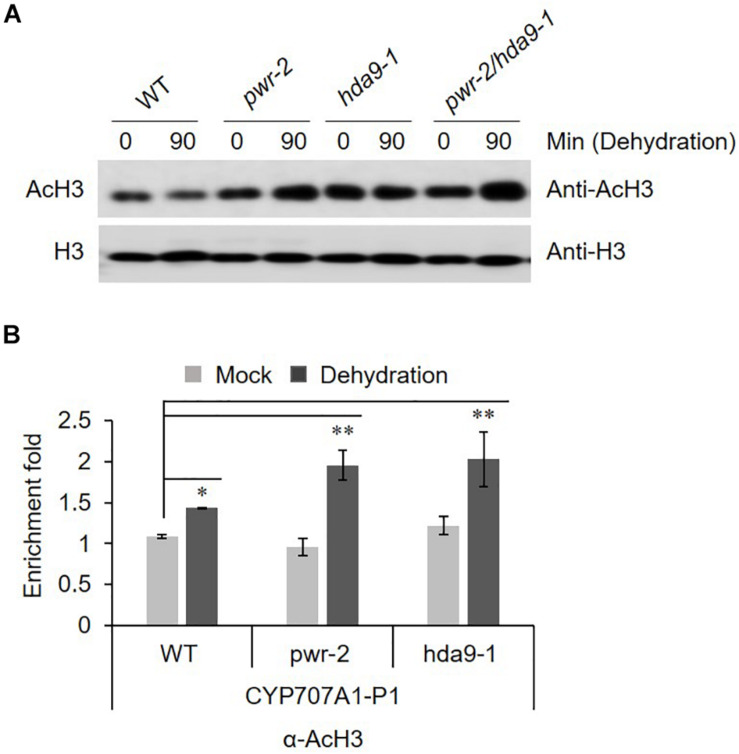
**(A)** AcH3 level is increased during drought stress. Nuclear protein was isolated from 2 weeks-old *Arabidopsis* WT (Col-0), *pwr-2*, *hda9-1*, and *pwr-2/hda9-1* seedlings after drought stress treatment for 0 or 90 min and then separated by SDS-PAGE. The anti-H3 band from a duplicate gel shows that similar amounts of proteins were loaded. **(B)** ChIP-qPCR assays of the *CYP707A1* promoter using antibody to acetylated histone 3 (AcH3). Chromatin from 2-week-old WT Col-0, *pwr-2* and *hda9-1* mutant plants subjected to drought treatment for 0 or 90 min was immunoprecipitated with anti-AcH3, and the amount of DNA in the immune complex was determined by qRT-PCR. Error bars representing SE (*n* = 3 independent experiments). Significant difference was determined by Student *t*-test (^∗^*p* < 0.05, ^∗∗^*p* < 0.01).

PWR and HDA9 together regulate the acetylation status of numerous genes, specifically at H3K9, H3K14, and H3K27. SANTb-domain proteins such as PWR preferentially bind to acetylated histone H3 but not H4. Moreover, it has been proposed that the SANTb domain of PWR defines the protein’s substrate specificity for binding to HDA9 (Kim et al., 2016). Therefore, we were interested to test the acetylation status of histone H3 in response to dehydration stress in *pwr* and *hda9* mutants. Compare to that in WT, the total AcH3 levels in the *pwr* and *hda9* single mutants as well as the *pwr/hda9* double mutant were increased by drought stress (Figure 6A). Since the total acetylation level of AcH3 was increased in *pwr* and *hda9* mutant, we assumed that the hyper-induction of *CYP707A1* gene expression in the *pwr* mutant (Figure 5) was largely due to hyperacetylation of the CYP707A1 promoter. The transcript levels of all four *CYP707A* genes increase in response to ABA and to abiotic stresses, including dehydration (Kushiro et al., 2004; Saito et al., 2004). To determine whether the increase in *CYP707A1* gene expression in *pwr* and *hda9* upon dehydration is due to chromatin remodeling, we carried out chromatin immunoprecipitation (ChIP) assays to assess the AcH3 level of *CYP707A1*. The level of AcH3 at one region of the *CYP707A1* promoter, P1 (Figure 6B), was higher in *pwr* mutant plants than in WT after 90-min dehydration stress, whereas we saw no differences at the P2 and P3 promoter regions (Supplementary Figures S4A,B). Interestingly, the level of AcH3 at the P1 region was also high in the *hda9* mutant after dehydration (Figure 6B). However, we detected no difference in AcH3 levels in the *CYP707A2* promoter during dehydration stress in the *pwr* mutant compared with the WT (Supplementary Figure S4C). Taken together, these results indicated that during drought stress, PWR and HDA9 repress the expression of *CYP707A1* through histone deacetylation to allow ABA accumulation, and that the P1 region is important for the activation or repression of *CYP707A1* during drought stress.

## Discussion

Posttranslational protein/histone modifications such as acetylation, methylation, phosphorylation, and ubiquitination play pivotal roles in plant growth and development, genome integrity, and stress responses. Histone acetylation and deacetylation, mediated by histone acetyltransferases (HATs) or histone deacetylases (HDACs), are reversible processes that promote or repress gene expression (Struhl, 1998). The RPD3/HDA1-type class 1 HISTONE DEACETYLASE 9 (HDA9), among the 18 histone deacetylases (HDACs) identified in *Arabidopsis*, interacts directly with PWR, a homolog of the human protein NCOR1 (Pandey et al., 2002; Kim et al., 2016). PWR, which was initially identified as being involved in regulating floral determinacy network, contains two important SANT domains known as SANTa and SANTb, which are required for its interaction with HDA9 and mediation of HISTONE (H3) deacetylation. PWR and HDA9 regulate several processes in *Arabidopsis*, including regulating flowering time by repressing *AGAMOUS-LIKE 19* (*AGL19*). PWR is known for its role in chromatin modification and regulation of developmental processes. Here, we have identified a previously unknown function of PWR regulating abiotic stresses.

### Physiological Effect of PWR on ABA and Drought Stress Signaling

PWR regulates plant growth and development, flowering time and the floral determinacy network. Moreover, PWR interacts with HDA9 and regulates flowering time in *Arabidopsis* by repressing *Agamous-Like 19* (*AGL19*) (Yumul et al., 2013; Kim et al., 2016). While HDA9 requires PWR for its nuclear transport and promoter association, and the HDA9-PWR-WRKY53 complex integrates and regulates multiple signaling pathways to mediate global gene expression responsible for leaf senescence (Chen et al., 2016). To explore the relationship between PWR and HDA9 during abiotic stress, we investigated the phenotypes of loss-of-function mutants of PWR (*pwr-2* and *pwr-3*) in the presence of ABA. Since HDA9 positively regulates plant response to ABA and dehydration (van Zanten et al., 2014; Kang et al., 2015), we expected that PWR might also play a positive role in ABA signaling and dehydration stress. We observed that *pwr-2* and *pwr-3* mutants were less sensitive than WT (Col-0) to ABA and impaired stomatal closure in the presence of exogenous ABA (Figure 1). Loss of PWR resulted in ABA-associated phenotypes such as ABA insensitivity in seed germination and post-germinative growth (Figure 2 and Supplementary Figure S2) and enhanced water loss, ultimately leading to drought sensitivity (Figure 3). Taken together, these data suggest that unlike HDA9, PWR is a positive regulator of ABA signaling. As previously reported, the phytohormone ABA quickly accumulates in response to stresses and plays a pivotal role in plant survival (Xiong et al., 2002, Xiong and Zhu, 2003). ABA is also a key regulator in stomatal movement that regulates water loss (Luan, 2002; Zhu, 2002). Indeed, ABA-related genes were suppressed in both *pwr* and *hda9* mutants (Figure 2E), indicating that PWR is a central regulator of ABA response. Since PWR and HDA9 physically interact and work together in the same complex (Kim et al., 2016), we tested the genetic interaction of PWR and HDA9. As expected, *pwr*, *hda9*, and *pwr*/*hda9* double mutants displayed the same insensitivity toward exogenously applied ABA and hypersensitivity to drought stress (Figures 2, 3). HDA9 binds to the active genes and may either prevent promiscuous cryptic gene expression or compete with other HDACs for binding to the same site. Transcriptome and ChIP-seq analyses have shown that HDA9 also binds the *PYL* gene promoter (Chen et al., 2016). These results indicated that PWR and HDA9 might work with specific transcription factors to repress their target genes during seed germination and drought stress.

### Powerdress and HDA9 Interact With ABI4 and Regulate Histone Acetylation During Drought Stress

HDA9 directly interacts with PWR, and the two proteins work together in the same repressor complex to regulate morphological and developmental processes in plants (Pandey et al., 2002; Kim et al., 2016). Given that the *pwr* and *hda9* mutants were insensitive to ABA and sensitive to drought stress (Figures 2, 3), we assessed the interactions of PWR and HDA9 with the components of the ABA signaling and drought stress pathways. PWR and HDA9 interacted with ABI4 along with ABI3 (Figure 4 and Supplementary Figure S3). ABI4 was initially identified on the basis of the insensitivity of *abi4* mutants to ABA, and later to salt and mannitol (Finkelstein, 1994; Quesada et al., 2000). ABI4 binds specifically to ABRE elements and regulates the expression of several genes in response to abiotic stresses (Mizoi et al., 2012). Notably, ABI4 is a versatile activator and a repressor of gene expression (Söderman et al., 2000; Nakabayashi et al., 2005; Koussevitzky et al., 2007; Bossi et al., 2009; Giraud et al., 2009; Reeves et al., 2011; Shu et al., 2018). ABI4 induces the expression of genes involved in seed dormancy and ABA signaling (Reeves et al., 2011), while repressing genes involved in ABA catabolism (*CYP707A* genes), ethylene biosynthesis (*ACS* genes), retrograde signaling (*AOX1a*), *ARR* genes, which are involved in cytokinin-induced degradation of *ABI5* (Finkelstein and Lynch, 2000; Lopez-Molina et al., 2001; Huang et al., 2017) and genes involved in fatty acid biosynthesis, photosynthesis, pigment and wax metabolic processes (Shu et al., 2013; Dong et al., 2016). These lines of evidence indicate that ABI4 both induces and represses target genes. We therefore tested the genes suppressed by ABI4 in loss-of-function mutants of PWR (*pwr-2* and *pwr-3*). In response to dehydration, the transcript levels of *CYP707A1*, *ACS4* and *AOX1a* in *pwr* mutants were significantly higher than those in WT (Figure 5), whereas there were no significant differences in the expression of *CYP707A2* and *CYP707A3* in *pwr* mutants as compared to WT. As PWR and HDA9 regulate the acetylation status of numerous genes specifically at H3K9, H3K14, and H3K27, we measured the total acetyl histone H3 (AcH3) levels in *pwr* and *hda9* mutants (Figure 6A). The AcH3 level was significantly increased in *pwr* and *hda9* mutants upon dehydration stress, suggesting that the total AcH3 increases in response to dehydration stress in the mutants. However, effects of the acetylation status at the specific promoter regions of target genes responsible for drought stress responses could not be excluded. Therefore, we analyzed the AcH3 level at the *CYP707A1* promoter and observed that after dehydration, the association of AcH3 was higher at the P1 region, but not the P2 or P3 regions, of the *CYP707A1* promoter in the *pwr-2* mutant compared to WT (Figure 6B and Supplementary Figure S4). Surprisingly, AcH3 association at the *CYP707A1* P1 region was also increased in the *hda9* mutant upon dehydration (Figure 6B). However, there was no difference in the *CYP707A2* promoter AcH3 level during dehydration stress in the *pwr* mutant compared to the WT (Supplementary Figure S4), suggesting that PWR and HDA9 are both required for repression of *CYP707A1* in response to drought stress.

**FIGURE 7:**
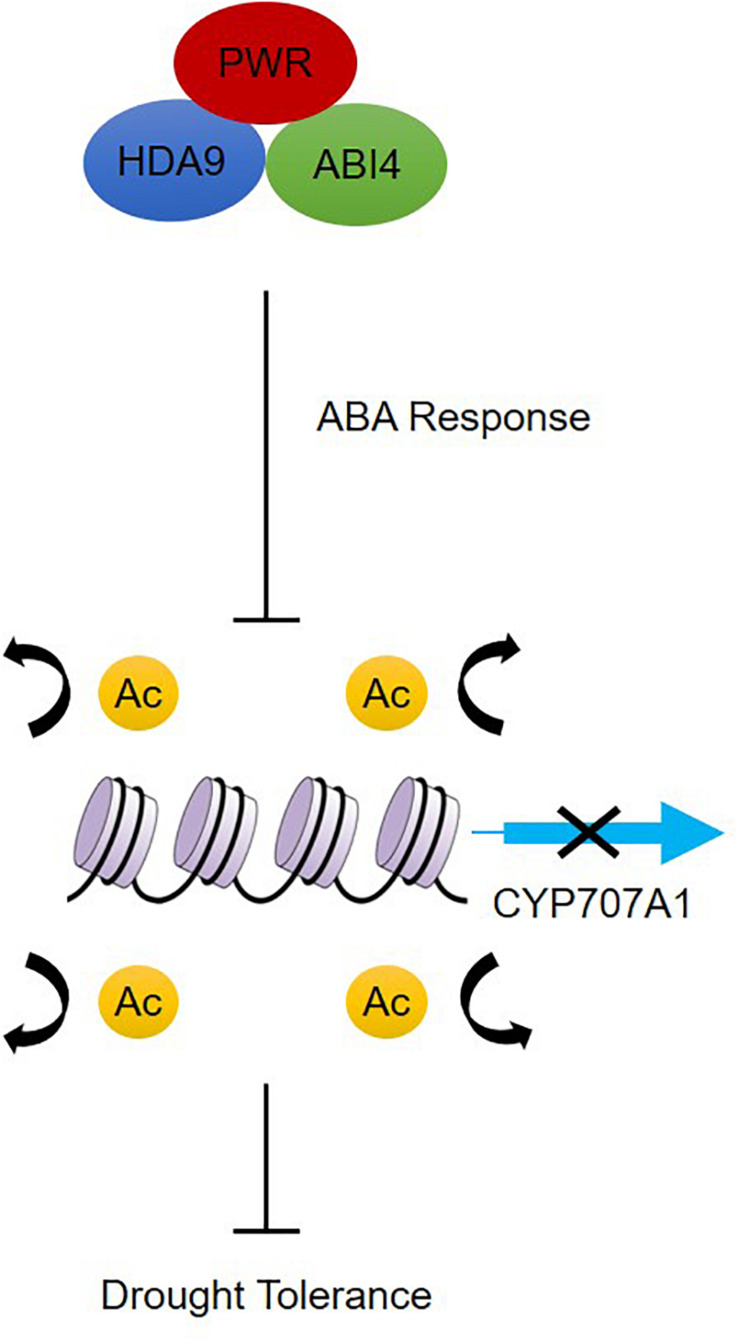
Proposed working model of PWR function in dehydration response. PWR together with HDA9 and ABI4 positively regulate ABA response either by acting at ABA receptors or by repressing negative regulators of the ABA pathway, which results in downstream expression of ABA-responsive genes. In the absence of dehydration stress, *CYP707A1* is expressed and regulates ABA hydroxylation, but dehydration stress triggers *PWR-HDA9-ABI4*-mediated repression of *CYP707A1* expression through the process of deacetylation and activates drought response.

Besides its role in regulating plant morphological and developmental processes, little is known about how PWR regulate signal transduction and chromatin modification in response to abiotic stresses. Based on our results, we propose a model for how PWR, together with HDA9 and ABI4, mediates biological functions by negatively regulating the expression of genes through chromatin modification during ABA-dependent drought stress (Figure 7). On one hand, PWR and HDA9 mutants displayed the same phenotype with and without exogenous ABA and interact specifically with ABI4, indicating that PWR, HDA9, and ABI4 might regulate the same set of genes in ABA pathway. On the other hand, in the absence of drought stress, ABA-catabolism-related genes such as *CYP707A1* are activated by histone acetylation; the *CYP707A1* gene product, (+)-abscisic acid 8′-hydroxylase, then converts active ABA to the inactive form (8′-hydroxy-ABA) inside guard cells, resulting in loss of turgor pressure and stomatal opening (Xiong et al., 2002, Xiong and Zhu, 2003). ABI4 was previously reported to suppress the *CYP707A* genes by directly binding to their promoters (Shu et al., 2013), and here we report that ABI4 together with PWR and HDA9 may associate with the *CYP707A1* promoter. This possible association with the PWR-HDA9-ABI4 repressor complex represses *CYP707A1* expression through histone deacetylation and results in drought tolerance. In addition to repressing genes, ABI4 is also involved in the activation of several genes. Since HDA9 and PWR regulate a wide range of genes involved in several key physiological processes including autophagy, pathogenesis and senescence, there might be some other unidentified genes through which HDA9 and PWR regulate ABA signaling and drought stress. Further study is required to uncover whether the PWR-HDA9-ABI4 complex is also involved in activating genes responsible for ABA signaling and drought tolerance.

## Data Availability Statement

The original contributions presented in the study are included in the article/Supplementary Material, further inquiries can be directed to the corresponding author.

## Author Contributions

IK, AA, and D-JY conceived and designed the experiments. IK, HK, SZ, MJ, DB, JP, and CL performed the experiments. IK, AA, JP, WK and D-JY analyzed the data and wrote the manuscript. All authors reviewed and approved the final manuscript.

## Conflict of Interest

The authors declare that the research was conducted in the absence of any commercial or financial relationships that could be construed as a potential conflict of interest.

## Footnotes

1 http://www.arabidopsis.info

2 http://www.arabidopsis.org
