# Supplementary material for: PWR/HDA9/ABI4 Complex Epigenetically Regulates ABA Dependent Drought Stress Tolerance in Arabidopsis
Source: Front Plant Sci. 2020 May 26;11:623. doi: 10.3389/fpls.2020.00623 (PMC7266079; doi:10.3389/fpls.2020.00623)
Supplement: Supplementary file 1 [file Data_Sheet_1.docx]

**Supplemental Information.** Supplemental information contains four Figures and one Table.


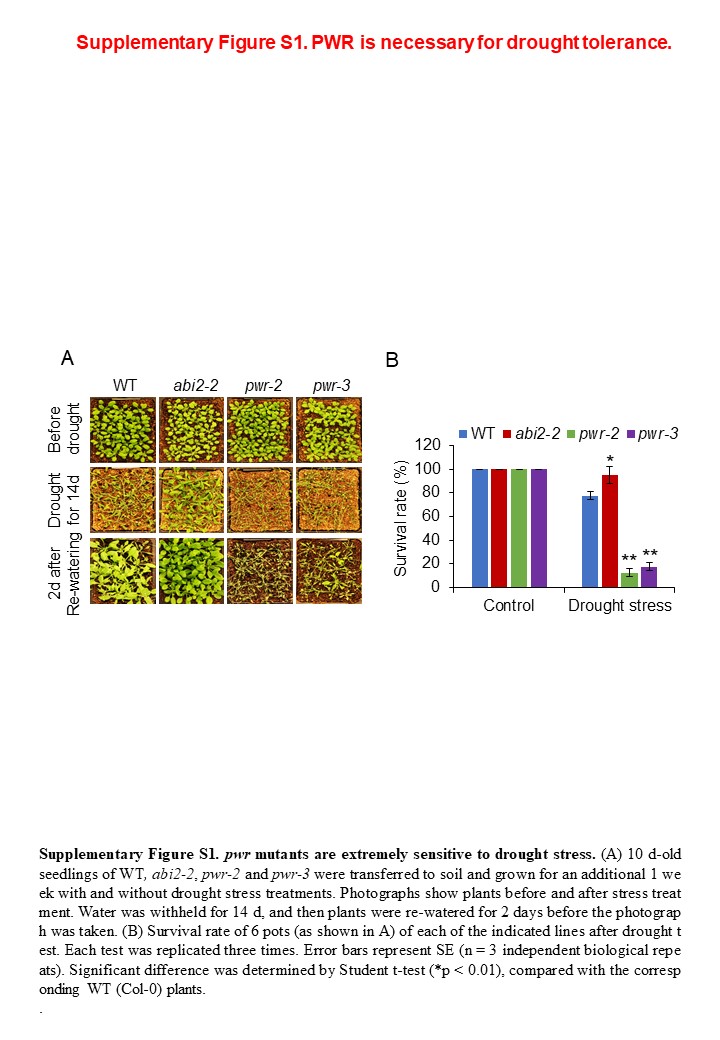


**Supplementary Figure S1*. pwr* mutants are extremely sensitive to drought stress.** (A) 3-week-old plants of genotypes WT (Col-0)*, abi2-2*, *pwr-2* and *pwr-3* were submitted to drought stress assay by withholding water for 14-day and subsequently rewatered after the drought period. Photographs show plants before and after stress treatment. (B) Survival rate of 6 pots (as shown in A) of each of the indicated lines after drought test. Each test was replicated three times. Error bars represent SE (n = 3 independent biological repeats). Signiﬁcant difference was determined by Student t-test (*p < 0.05, **p < 0.01).


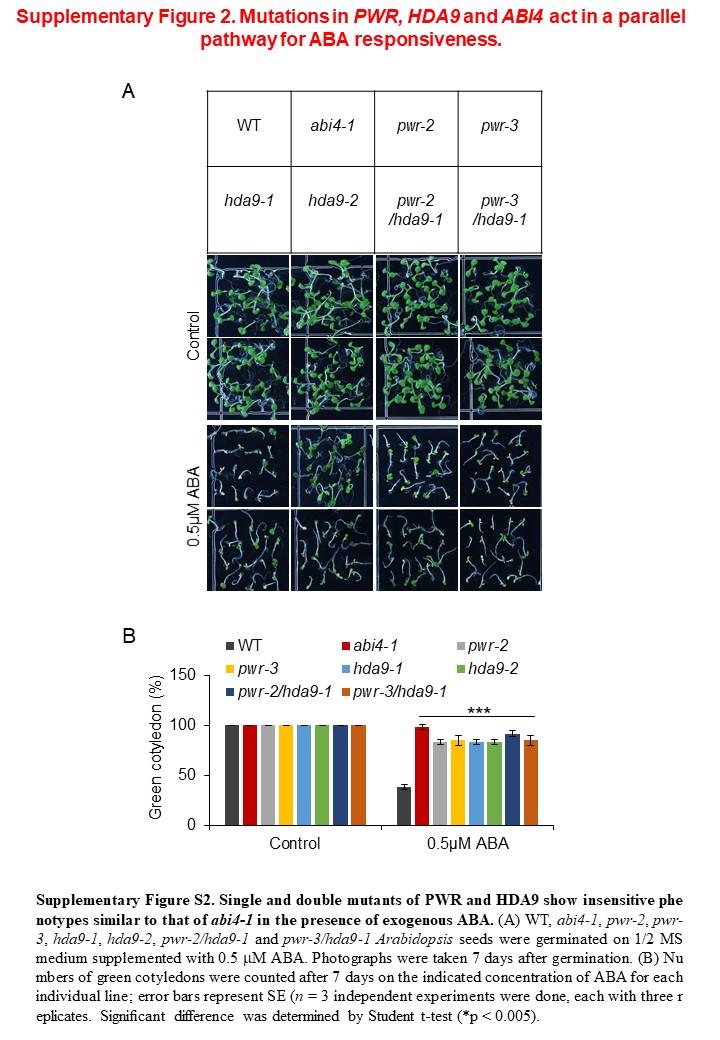


**Supplementary Figure S2. Single and double mutants of PWR and HDA9 show insensitive phenotypes similar to that of *abi4-1* in the presence of exogenous ABA.** (A) WT, *abi4-1*, *pwr-2*, *pwr-3*, *hda9-1*, *hda9-2*, *pwr-2/hda9-1* and *pwr-3/hda9-1* *Arabidopsis* seeds were germinated on 1/2 MS medium supplemented with 0.5μM ABA. Photographs were taken 7 days after germination. (B) Numbers of green cotyledons were counted after 7 days on the indicated concentration of ABA for each individual line; error bars represent SE (*n* = 3 independent experiments were done, each with three replicates. Signiﬁcant difference was determined by Student t-test (***p < 0.005).


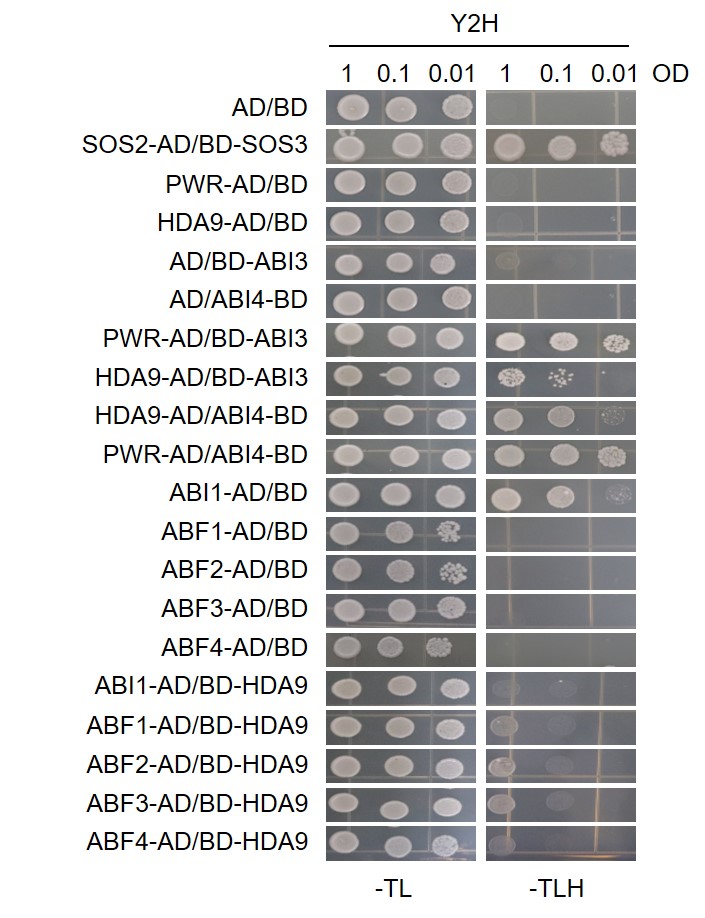


**Supplementary Figure S3. Screening of ABA transcription factors interacting with PWR and HDA9.** Screening of PWR and HDA9 interaction with ABA transcription factors using yeast two-hybrid assay. BD, *pDEST32* (bait plasmid); AD, *pDEST22* (prey plasmid). The co-transformed yeast strains were plated on the control (-TL) and selective media (-TLH). The combinations with empty plasmid were used as negative controls and *SOS2*-AD/*SOS3*-BD was used as positive control.


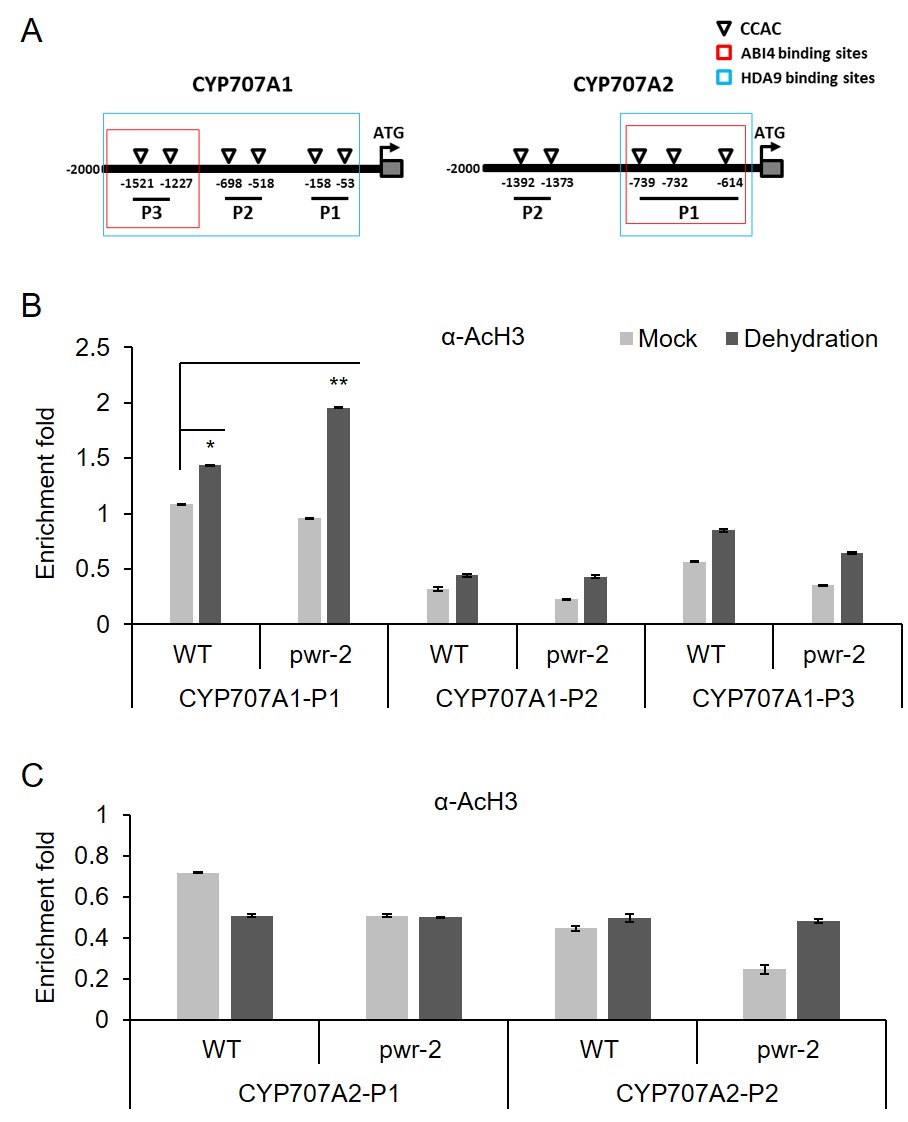


**Supplementary Figure S4. Histone H3 acetylation of the *CYP707A1* and *CYP707A2* promoters in *pwr* and *hda9* mutant plants during drought stress.** (A) Structures of the *CYP707A1* and *CYP707A2* promoters. (B-D) ChIP-qPCR assays of the *CYP707A1* (B, C) and *CYP707A2* (D) promoters using antibody to acetylated histone 3 (AcH3). Chromatin from 2-week-old WT Col-0, *pwr-2* and *hda9-1* mutant plants subjected to drought treatment for 0 or 90min was immunoprecipitated with anti-AcH3, and the amount of DNA in the immune complex was determined by qRT-PCR. Error bars representing SE (n = 3 independent experiments). Signiﬁcant difference was determined by Student t-test (*p < 0.05, **p < 0.01).

**Supplementary Table S1. Primer used in the study.**

| **Primers** | **Sequence (5' to 3')** | **Purpose** |
| --- | --- | --- |
| GABI-Kat LB | Cccatttggacgtgaatgtagacac | Genotyping |
| PWR F (P3) | Tcccggggatgccgcaggatcacgcttcgt |  |
| PWR R (P4) | Acgctccattgagatgttatcg |  |
| P1(hda9 genotyping F) | Atggcctgcatagcaagatg |  |
| P2(hda9 genotyping R) | Aaaaatccggcgcaaaatac |  |
| LBb1.3 | Attttgccgatttcggaac |  |
| ACS4 qRT-F | Acatgagacctctccttaga | qRT-PCR |
| ACS4 qRT-R | ccagttagagacatttgaca |  |
| CYP707A1 qRT-F | tcatctcaccaccaagta |  |
| CYP707A1 qRT-R | aaggcaattctgtcattcta |  |
| CYP707A2 qRT-F | atccatcactcctccgaattcttcc |  |
| CYP707A2 qRT-R | tccatttccgaatggcatgtacg |  |
| CYP707A3 qRT-F | catgccttttggtagtgggattcat |  |
| CYP707A3 qRT-R | cggcccatactgaattccatcg |  |
| AOX1a qRT-F | ctcttcgttggcctaccgatt |  |
| AOX1a qRT-R | aaccattccaggtactgctgctac |  |
| Lhcb1.1 qRT-F | ttgaaggctacagagtcgcaggaa |  |
| Lhcb1.1 qRT-R | accagtgacgatggcttgaacg |  |
| TUB8 qRT-F | cgtggatcacagcaatacagagcc |  |
| TUB8 qRT-R | cctccgcacttccacttcgtcttc |  |
| attB1 adapter | ggggacaagtttgtacaaaaaagcaggct | Plasmid construction,  LCI, Y2H, Co-IP |
| attB2 adapter | ggggaccactttgtacaagaaagctgggt |  |
| PWR attB1 | aaaaaagcaggcttcatgccgcaggatcacgcttcgtgg |  |
| PWR attB2 | agaaagctgggtctcacgtggctgcctctgctacacca |  |
| PWR no attB2 | agaaagctgggtccgtggctgcctctgctacacca |  |
| HDA9 attB1 | aaaaaagcaggcttaatgcgttccaaggacaaaatctc |  |
| HDA9 attB2 | agaaagctgggtcttatgacgcatcgttatcgttgtc |  |
| HDA9 no attB2 | agaaagctgggtctgacgcatcgttatcgttgtctcc |  |
| ABI4 attB1 | aaaaaagcaggcttaatggaccctttagcttccca |  |
| ABI4 attB2 | agaaagctgggtcttaatagaattccccca |  |
| ABI4 no attB2 | agaaagctgggtcatagaattccccca |  |
| CYP707A1-P1- Fw | ccttcacatctcccacttgt | ChIP primers |
| CYP707A1-P1-Rev | gtgagaaacaaggcggagat |  |
| CYP707A1-P2-Fw | tcccactcttttattcactc |  |
| CYP707A1-P2-Rev | aaatgtggggtaaagtctac |  |
| CYP707A1-P3-Fw | aaagatgatgaggattcggt |  |
| CYP707A1-P3-Rev | gtaaaatgaagatgtgtggc |  |
| CYP707A2-P1-F | cgtggatttctagggatgtc |  |
| CYP707A2-P1-R | cttcatcatatcttggacct |  |
| CYP707A2-P2-F | aagtgtagtgtggggttagc |  |
| CYP707A2-P2-R | cgcagtactatttatgtggt |  |
| TUB4-F | cgagaggatcacagcaatacag |  |
| TUB4-R | ggatccattccacaaagtagga |  |
